# Functional Characterization of Ao4g24: An Uncharacterized Gene Involved in Conidiation, Trap Formation, Stress Response, and Secondary Metabolism in *Arthrobotrys oligospora*

**DOI:** 10.3390/microorganisms12081532

**Published:** 2024-07-26

**Authors:** Lirong Zhu, Meichen Zhu, Xuemei Li, Yanmei Shen, Shipeng Duan, Jinkui Yang

**Affiliations:** State Key Laboratory for Conservation and Utilization of Bio-Resources in Yunnan, Key Laboratory for Southwest Microbial Diversity of the Ministry of Education, and School of Life Science, Yunnan University, Kunming 650032, China; zhulirong_dif5@stu.ynu.edu.cn (L.Z.); zmc201789@163.com (M.Z.); xmli@mail.ynu.edu.cn (X.L.); shenyanmei@stu.ynu.edu.cn (Y.S.); duanshipeng@stu.ynu.edu.cn (S.D.)

**Keywords:** nematode-trapping fungus, unknown gene, sporulation, cellular process, nematode-trapping ability

## Abstract

*Arthrobotrys oligospora* is a typical nematode-trapping (NT) fungus, which can secrete food cues to lure, capture, and digest nematodes by triggering the production of adhesive networks (traps). Based on genomic and proteomic analyses, multiple pathogenic genes and proteins involved in trap formation have been characterized; however, there are numerous uncharacterized genes that play important roles in trap formation. The functional studies of these unknown genes are helpful in systematically elucidating the complex interactions between *A. oligospora* and nematode hosts. In this study, we screened the gene AOL_s00004g24 (*Ao4g24*). This gene is similar to the SWI/SNF chromatin remodeling complex, which was found to play a potential role in trap formation in our previous transcriptome analysis. Here, we characterized the function of *Ao4g24* by gene disruption, phenotypic analysis, and metabolomics. The deletion of *Ao4g24* led to a remarkable decrease in conidia yield, trap formation, and secondary metabolites. Meanwhile, the absence of *Ao4g24* influenced the mitochondrial membrane potential, ATP content, autophagy, ROS level, and stress response. These results indicate that *Ao4g24* has crucial functions in sporulation, trap formation, and pathogenicity in NT fungi. Our study provides a reference for understanding the role of unidentified genes in mycelium growth and trap formation in NT fungi.

## 1. Introduction

Nematode-trapping (NT) fungi are a type of carnivorous microorganism that use mycelia to form specialized trapping devices known as traps (such as adhesive knobs, adhesive networks, and constricting rings) to capture, kill, and consume nematodes [1]. As a representative NT fungus, *Arthrobotrys oligospora* has been widely studied to show the interaction between NT fungi and nematodes. *A. oligospora* can produce adhesive networks (traps) and secret serine proteases to capture and digest nematodes [2,3]. These traps serve as an important tool to kill nematodes and are a symbol of the lifestyle transition from saprotrophic to predatory. Based on the genomic and proteomic analyses of *A. oligospora*, multiple cellular processes, such as the cell cycle, cytoskeleton adhesion, signal transduction, peroxisomes, and energy metabolism, have been shown to be involved in trap formation [4]. Previous studies have shown that multiple genes related to signaling pathways, such as G-protein, Mitogen-Activated Protein Kinase (MAPK), and cAMP-Dependent Protein Kinase (cAMP-PKA), are essential for trap formation [5]. In addition, several peroxin and autophagy-related genes are reported to play essential roles in trap formation in *A. oligospora* [6,7,8,9]. Despite the increasing evidence indicating that multiple cellular processes perform crucial roles in trap formation, there are numerous uncharacterized genes that are also important for trap formation in NT fungi. Functional studies of these unknown genes will provide a comprehensive and in-depth reference for understanding the regulatory mechanism of trap formation in NT fungi.

In this study, we found multiple genes related to chromatin organization that were engaged in the trap formation process demonstrated in our previous transcriptome analysis. The expression of an uncharacterized gene, AOL_s00004g24 (*Ao4g24*), which is similar to the SWI/SNF chromatin remodeling complex, was significantly upregulated during trap formation (Figure S1). To investigate the role of *Ao4g24*, the phylogenetic and conserved functional domains of the Ao4g24 homologous proteins in filamentous fungi (including *Magnaporthe oryzae*, *Fusarium graminearum*, *Beauveria bassiana*, *Aspergillus niger*, and *Aspergillus nidulans*) and NT fungi (*Arthrobotrys flagrans*, *Dactylellina cionopaga*, and *Dactylellina haptotyla*) were analyzed. Our results showed that the *Ao4g24* gene is specific to NT fungi. However, using multiple sequence alignment, it was shown that it has a similar amino acid sequence to Snf5 of the SWI/SNF chromatin remodeler in other filamentous fungi. Therefore, it is justifiable to hypothesize that *Ao4g24* performs a critical role in the trap formation of *A. oligospora*.

The SWI/SNF (switching defective/sucrose non-fermenting) class of the ATP-dependent chromatin remodeling complex is conserved among eukaryotes and has essential roles in regulating chromatin architecture and gene expression [10]. The SWI/SNF complex was first discovered in *Saccharomyces cerevisiae* and contains 12 subunits and functions to regulate the nucleosome structure for chromatin remodeling [11]. There is increasing evidence that the SWI/SNF complex has multiple functions in fungi, including functions in regulating hyphal differentiation, sporulation, stress response, virulence, and pathogenicity [12,13,14]. In *S. cerevisiae*, *Scsnf5* helps the core ATPase catalytic subunit Snf2 of the SWI/SNF complex bind to nucleosome DNA [15]. The *Scsnf5* mutant strains lose the ability to utilize galactose and glycerol, display hypersensitivity to lithium ions, and show defects in the process of activating the transcription of *Scsuc2* in *S. cerevisiae* [16,17]. In *Candida albicans*, Snf5 is essential for maintaining mycelial development, metabolic homeostasis, and virulence [18,19]. In in-depth studies, several SWI/SNF family genes were confirmed to be essential for reprogramming gene expression, such as hyphal growth, stress response, and metabolism [12,19,20]. In *C. albicans*, *snf2* associates with *swi1* (core subunit of SWI/SNF complex) and promotes true mycelial growth [12], and the inactivation of *snf6* leads to increased sensitivity to high temperature [21]. In *Candida glabrata*, the *snf6* mutants showed decreased utilization of carbon sources [22]. In yeast, *swi3* is important for regulating mitochondrial respiration and oxygen consumption [23]. In *Schizosaccharomyces pombe*, *Spsnf21* (orthologue of *snf2* in *S. cerevisiae*) plays an essential role in cell viability by regulating the chromatin centromere [24]. Combined with these studies, various subunits of the SWI/SNF complex perform a crucial role in maintaining gene expression, including hyphal development, conidiation, and pathogenicity in fungi. Despite the multi-cellular processes in the regulation of hyphal development and pathogenicity by the SWI/SNF chromatin remodeler, the biological role of *Ao4g24* in NT fungi and the underlying mechanism remain unexplored.

Here, we characterized the role of the unknown gene *Ao4g24* in the NT fungus *A. oligospora* by performing gene knockout, multi-phenotypic comparison, and metabolome analysis. We aimed to reveal the function of *Ao4g24* in the mycelial growth and development of *A. oligospora*. Our results provide important evidence regarding the regulation of the novel gene *Ao4g24*, which is similar to the SWI/SNF chromatin remodeler for conidia generation and trap formation in *A. oligospora*.

## 2. Materials and Methods

### 2.1. Strains and Culture Conditions

*A. oligospora* (ATCC24927) and Δ*Ao4g24* mutant strains were cultured on potato dextrose agar (PDA) medium at 28 °C for 5–7 days. The knockout vector PRS426 and plasmid pCSN44 encoding the selective label hygromycin were preserved using the *Escherichia coli* strain DH5α (Tsingke, Beijing, China). To select the recombinant transformants, the *S. cerevisiae* strain FY834 was cultivated in yeast extract peptone dextrose (YPD) broth at 30 °C. The nematode *Caenorhabditis elegans* N2 strain was grown on oatmeal medium at 26 °C for 14 days and employed to stimulate trap formation.

### 2.2. Bioinformatic Analysis of Ao4g24

The amino acid sequence of Ao4g24 (AOL_s00004g24) was downloaded from the NCBI database, and the homologous proteins from several different fungi were retrieved through BLAST comparison. The ClustalW software (version 2.0) was used to execute multiple sequence alignments. The sequence similarity of Ao4g24 and other homologous proteins was analyzed by DNAman software (version 6.0.3.99). The conserved domain of Ao4g24 was predicted using IntroProScan and visualized by Tbtools (version 2.096). MEGA X was used to construct a neighbor-joining tree, and the bootstrap values were determined based on 1000 replicates. The isoelectric point and molecular weight of Ao4g24 were calculated using the pI/MW online website.

### 2.3. Gene Deletion and Verification

As previously mentioned, the homologous recombination technique was used to create the mutant strains of *Ao4g24* [25]. The two homologous fragments of *Ao4g24* were amplified via a PCR reaction using the specific primers (Table S1). Plasmid PCSN44 served as a template for the amplification of the hygromycin tolerance gene (*hph*). The above three purified fragments were inserted into the linearized pRS426 plasmid to construct a fusion vector (*Ao4g24*-*hph*-pRS426). The pure recombinant vector was converted into the protoplasts of *A. oligospora* following a previously described protocol [25,26]. The transformants were initially cultured on PDAS medium supplemented with 200 μg/mL of hygromycin B and confirmed using PCR. The positive transformants were then further validated through the real-time quantitative PCR (RT-qPCR). Table S1 lists the primers used to verify the positive transformants.

### 2.4. Hyphal Growth and Sporulation Comparison

The colonies of the WT and Δ*Ao4g24* mutant strains with the same diameter were cultivated on PDA medium for 3–5 days and then individually transferred into PDA, TG, and TYGA media at 28 °C for 5 days to observe mycelial morphology and calculate the growth rates. The colony diameters were recorded every 24 h. Calcofluor white (CFW) and 4′,6′-diamidino-2-phenylindole (DAPI) were used to visualize the hyphal septa and cell nuclei, respectively [27]. Following 14 days of incubation on CMY medium at 28 °C, the WT and mutant strains were harvested for conidia statistics by eluting the spores with 15 mL of sterile distilled water. Then, 2 × 10^4^ conidia were coated on WA medium and cultivated for 4 h, 8 h, and 12 h to count spore germination [28].

### 2.5. Analyses of Trap Formation and Pathogenicity

To analyze the trap formation, spore suspensions containing 2 × 10^4^ conidia were coated on WA plates at 28 °C for 2–3 days. Subsequently, each WA plate was inoculated with about 400 nematodes (*C. elegans* N2) to stimulate trap production. Then, we observed the trap morphology and calculated the trap counts and nematode death rate at 12 h intervals [29]. Equal-sized fungal colony discs were inoculated into PD broth medium containing nematode extract at 180 rpm and 28 °C for a duration of 5 days. The fermentation liquor was collected by filtering the mycelia. The protease activity was qualitatively evaluated by adding the equivalent fermentation fluid to the casein skimmed milk plates. It was then quantitatively analyzed as described above, using a BCA protein quantitative kit (Dingguo, Beijing, China) [30].

### 2.6. Stress Tolerance Analysis

In order to detect the sensitivity of *Ao4g24* to various environmental stress reagents, fungal discs (7 mm in diameter) from the WT and Δ*Ao4g24* mutant strains were inoculated on TG medium containing different concentrations of stress reagents, including oxidative reagents (0.05–0.09 mM menadione and 5–15 mM H_2_O_2_) and osmotic reagents (0.1–0.2 M NaCl and 0.25–0.5 M sorbitol), and subjected to different temperature conditions (34 °C–42 °C). Following 6 days of incubation at 28 °C, the colony diameters were calculated. The sensitivity of the mutant strains to the stress reagents was determined by calculating the relative growth inhibition rate (RGI). All the tests were performed in triplicate.

### 2.7. Observation of Autophagy and Cell Apoptosis

To observe the autophagosomes, 20 μL of 10 μg/mL MDC dye was used to stain the fresh mycelia of the WT and mutant strains for 10 min after they were cultivated on PDA medium for 5 days. The samples were then observed under a fluorescence microscope. The DNA damage and cell apoptosis were evaluated using a TUNEL assay kit. Propyl iodide (PI) and FITC-dUTP were used to stain the mycelial nuclei and DNA fragments, respectively. An indicator of the apoptosis level was the green/red fluorescence intensity ratio (FI) [8]. The internal structure of the mycelia was observed using a transmission electron microscope (TEM).

### 2.8. Evaluation of Mitochondrial Function and ROS Level

The mitochondrial morphology of the WT strain and Δ*Ao4g24* mutants was observed by staining with 20 μL of MitoTracker Green at a concentration of 10 μg/mL for 20 min. Then, the mitochondrial membrane potential (MMP) was stained with 20 μL of ethyl benzene and imidazole iodide carbon cyanide cyanine (JC-1) dye for 30 min, and the green/red FI values were evaluated. The ROS levels of the WT and mutant strains were evaluated using DHE (MCE, Shanghai, China) staining, and the FI was determined using Image J. Furthermore, the ROS level of the fresh hypha cultured on PDA medium for 3 days was stained with nitro tetrazolium blue chloride (NBT) (Solarbio, Beijing, China) for 40 min in the dark, washed twice through a tissue decolorization solution, and photographed after drying.

### 2.9. RT-qPCR Analysis

For the RT-qPCR analysis, mycelial samples were obtained at 3, 5, and 7 days on TYGA medium at 28 °C. A total RNA extraction kit (Axygen, Suzhou, China) was used to separate the total RNA from each sample [8], and cDNA was synthesized by reverse transcription using the PrimeScript RT kit (TaKaRa, Kusatsu, Japan). The LightCycler 480 SYBR green I master mix (Roche, Basel, Switzerland) was used to measure the transcriptional levels of the genes related to sporulation, stress response, and autophagy, and the β-tubulin gene served as an internal control. Table S2 shows the primers used for RT-qPCR analysis. The gene transcription level was calculated utilizing the 2^−∆∆CT^ method [31].

### 2.10. Metabolomics Analyses

Using a vacuum filtration pump, the fermentation liquor and mycelia were isolated after 7 days of inoculation in 250 mL of PD broth medium at 28 °C in a 180 rpm shaker. Subsequently, the fermentation broth was ultrasonicated for 40 min after being combined with ethyl acetate in a 1:1 *v*/*v* ratio. After standing for 12 h, the top extract was concentrated with a rotary evaporator, and the resulting precipitate was then dissolved using chromatography-grade methanol that was proportional to the mycelial sample weight. The dissolved sample was passed through a 0.22 μm membrane filter twice for HPLC-MS analysis [32]. The metabolic profile was analyzed using Thermo Xcalibur software version 3.0 (Thermo Fisher Scientific, Miami, OK, USA) and Compound Discoverer 3.0 software (Thermo Fisher Scientific) was used to analyze the non-targeted metabolomics [28].

### 2.11. Statistical Analysis

The trials were replicated three times, and the results were presented as the mean ± standard deviation (SD). The statistical software Graphad Prism (version 8.0) was used to conduct univariate analysis. Statistical significance was defined as a *p*-value below 0.05.

## 3. Results

### 3.1. Sequence and Phylogenetic Characterization of Ao4g24

**Figure 1:**
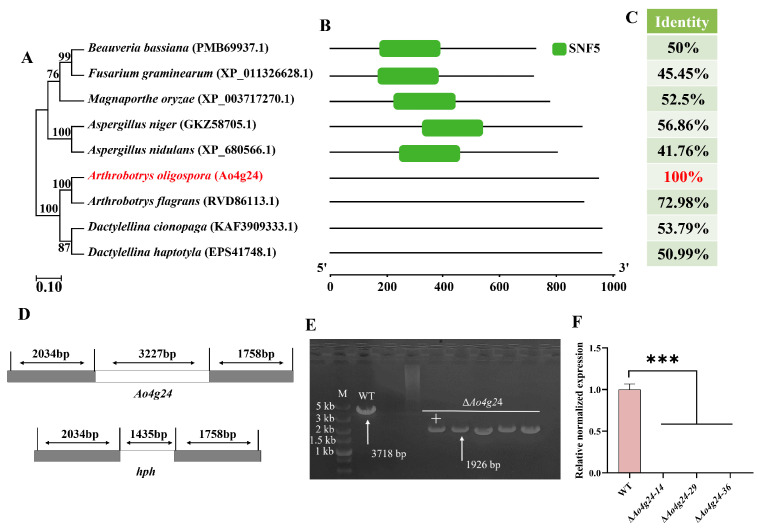
Phylogenetic analysis and validation of Δ*Ao4g24* mutant strains. (**A**–**C**) Phylogenetic (**A**), conserved domain (**B**), and sequence similarity (**C**) analyses of Ao4g24 orthologs from different fungi. (**D**) Diagram of *Ao4g24* knockout via homologous recombination. (**E**,**F**) Verification of positive transformants by PCR amplification (**E**) and RT-qPCR (**F**). M: DNA marker. A significant difference is denoted by an asterisk (*** *p* < 0.001).

In our transcriptome analysis, the transcription of *Ao4g24* was highly upregulated in the trap formation stage (24 h) (Figure S1). Bioinformatic analysis was conducted to predict the properties of *Ao4g24* in *A. oligospora*. Ao4g24 was found to have 943 amino acid residues, with a molecular weight of 103.53 kDa and expected isoelectric points of 4.63. According to the multiple sequence alignment conducted using Jalview software (version 2.11.3.3), Ao4g24 contains an amino acid sequence that is similar to that of the SNF5 domain of the SWI/SNF complex (Figure S2). The phylogenetic tree revealed that the homologs of Ao4g24 from different filamentous fungi were grouped into two distinct clades (Figure 1A). The homologous proteins of Ao4g24 from different NT fungal species formed a separate evolutionary branch. The conserved domain of Ao4g24 was not identified in *A. oligospora* (Figure 1B). The *A. oligospora* homologs exhibited a high level of sequence similarity with orthologs from other NT fungi, such as *A. flagrans* (72.98%), *D. cionopaga* (53.79%), and *D. haptotyla* (50.99%), but they had average sequence similarity with other filamentous fungi, including *A. nidulans* (41.76%) and *F. graminearum* (45.45%) (Figure 1C). In order to investigate the roles of *Ao4g24* in *A. oligospora*, we disrupted the gene via homologous recombination. Through PCR and RT-qPCR verification, we successfully obtained three Δ*Ao4g24* mutants (Figure 1E,F).

### 3.2. Ao4g24 Is Dispensable for Hyphal Growth

**Figure 2:**
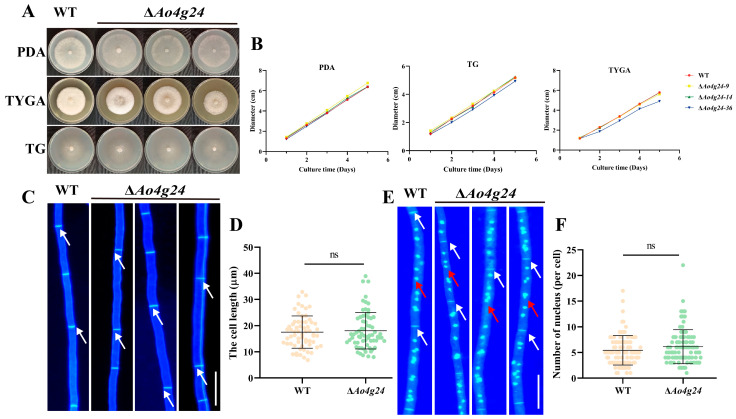
Comparison of mycelial growth, cell lengths, and nuclei counts in WT and Δ*Ao4g24* mutants. (**A**) WT and Δ*Ao4g24* mutants were grown on PDA, TG, and TYGA media at 28 °C for a duration of 5 days. (**B**) Growth rate measurement. (**C**) CFW staining of mycelia between WT and Δ*Ao4g24* mutants. Bar = 10 µm. The hyphal septa are denoted by white arrows. (**D**) Comparison of the length of hyphal septa between WT and Δ*Ao4g24* mutants. (**E**) The cell nuclei of WT and Δ*Ao4g24* mutants were stained using CFW and DAPI solution. Bar = 10 µm. The hyphal septa are denoted by white arrows, whereas the red arrows indicate the nuclei. (**F**) Calculation of nuclei counts in each cell, ns indicates there is no significant difference between WT and Δ*Ao4g24* mutants.

The mycelial morphology of the WT and Δ*Ao4g24* mutants showed no significant differences in PDA, TG, and TYGA media (Figure 2A). While the Δ*Ao4g24* mutants had a slower growth rate on TYGA medium compared to that of WT, no differences were observed in the PDA and TG media (Figure 2B). Furthermore, CFW and DAPI staining revealed that there were no differences between the Δ*Ao4g24* mutant strains and WT in terms of mycelial cell length and nucleus number (Figure 2C–F).

### 3.3. Ao4g24 Is Crucial for Conidiation

**Figure 3:**
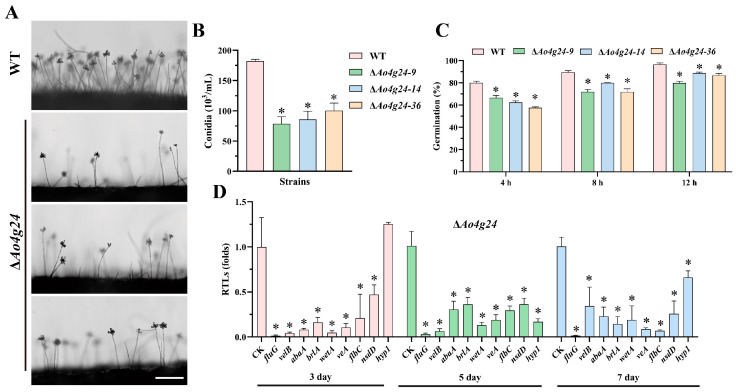
Analyses of conidia yield and transcription levels of genes related to sporulation in WT and Δ*Ao4g24* mutants. (**A**) Observation of conidiophores. Bar = 50 μm. (**B**) Conidia yields. (**C**) The spore germination rate. (**D**) Relative transcription levels (RTLs) of sporulation-related genes at 3, 5, and 7 days. CK is an established norm (RTL = 1) for statistical analysis. A significant difference is denoted by an asterisk (* *p* < 0.05).

Observations of the conidiophores and conidia yields revealed that the ∆*Ao4g24* mutant strains had fewer and sparser conidiophores relative to the WT strain (Figure 3A). In concordance with this, the disruption of *Ao4g24* led to a significant decrease in conidia yield. The WT strain produced approximately 1.8 × 10^5^ conidia/mL, whereas the conidia yield of ∆*Ao4g24* mutant strains was approximately 8.0 × 10^4^ conidia/mL (Figure 3B). Moreover, the spore germination rates in the ∆*Ao4g24* mutants at 4 h, 8 h, and 12 h were also considerably decreased compared to those of the WT strain (Figure 3C). In addition, the expression levels of eight genes related to sporulation, such as the *fluG*, *velB*, *abaA*, *brlA*, *wetA*, *veA*, *flbC*, and *nsdD* in the ∆*Ao4g24* mutant strains, were significantly downregulated at 3, 5, and 7 days on TYGA medium (Figure 3D).

### 3.4. Ao4g24 Is Required for Trap Formation and Pathogenicity

**Figure 4:**
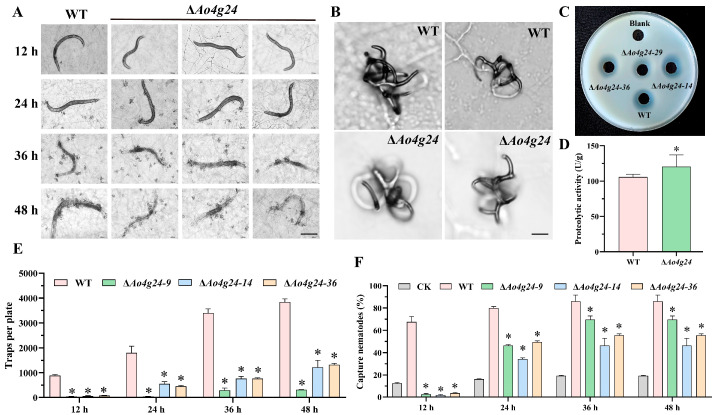
Comparison of WT and mutant strains for trap formation and pathogenicity. (**A**) Trap formation at different time points. Bar = 50 µm. (**B**) Trap morphology at 24 h. Bar = 10 μm. (**C**) Comparison of the extracellular proteolytic activity. (**D**) Quantitative analysis of extracellular protease activity. (**E**) Trap counts. (**F**) Nematode mortality. A significant difference is denoted by an asterisk (* *p* < 0.05).

Equal amounts of nematodes were added to the mycelia-filled WA plate to stimulate trap production. The number of traps induced by nematodes in the ∆*Ao4g24* mutant strains was remarkably decreased (Figure 4E). Additionally, the nematode predation efficiency in the WT strain was noticeably higher than that of the ∆*Ao4g24* mutant strains at four different time points (12–48 h) (Figure 4F). However, the loss of the *Ao4g24* gene (115 U/g) led to a slight increase in proteolytic activity relative to the WT strains (100 U/g) (Figure 4C,D). These results showed that *Ao4g24* is required for trap formation and nematode-capturing efficiency in *A. oligospora*.

### 3.5. Ao4g24 Regulates Autophagy and Mitochondrial Morphology

**Figure 5:**
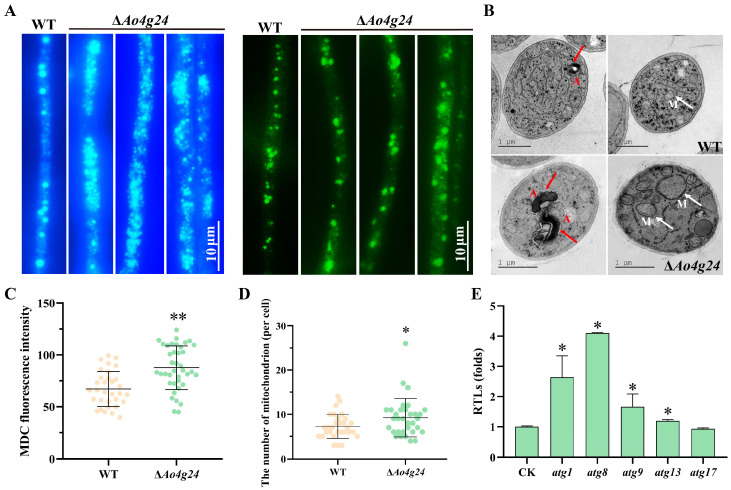
Comparison of autophagy and mitochondrial morphology. (**A**) Autophagosomes and mitochondria of WT and Δ*Ao4g24* mutant strains were stained with MDC and MitoTracker Green dye, respectively. Bar = 10 μm. (**B**) Observation of autophagosomes and mitochondria in TEM images. A: autophagosome; M: mitochondria; red arrows: autophagosome; white arrows: mitochondria. (**C**) Statistical analysis of MDC FI. (**D**) Statistical analysis of mitochondrial amounts in at least 30 TEM fields. (**E**) RTLs of autophagy-related genes. A significant difference is denoted by an asterisk (* *p* < 0.05, ** *p* < 0.01).

MDC solution was used to observe the autophagic process of mycelia in the Δ*Ao4g24* mutants. The Δ*Ao4g24* mutant strains showed a significantly increased accumulation of autophagosomes (Figure 5A), and the FI was also remarkably enhanced compared with that of the WT strain (Figure 5C). Furthermore, the expression levels of the autophagy-related genes (*atg1*, *atg8*, *atg9*, and *atg13*) were markedly increased relative to the WT strain (Figure 5E). In addition, the TEM images also revealed a notable increase in autophagy in the Δ*Ao4g24* mutant strains (Figure 5C). In order to examine the potential involvement of *Ao4g24* in energy maintenance, we analyzed the mitochondrial morphology using MitoTracker Green dye. As revealed by the hyphal staining and imaging, the mitochondrial volume became partially enlarged in the Δ*Ao4g24* mutant strains (Figure 5A). The TEM imaging analysis showed a statistically significant increase in the number of mitochondria (Figure 5D).

### 3.6. Ao4g24 Contributes to Cell Apoptosis and Mitochondrial Activity

**Figure 6:**
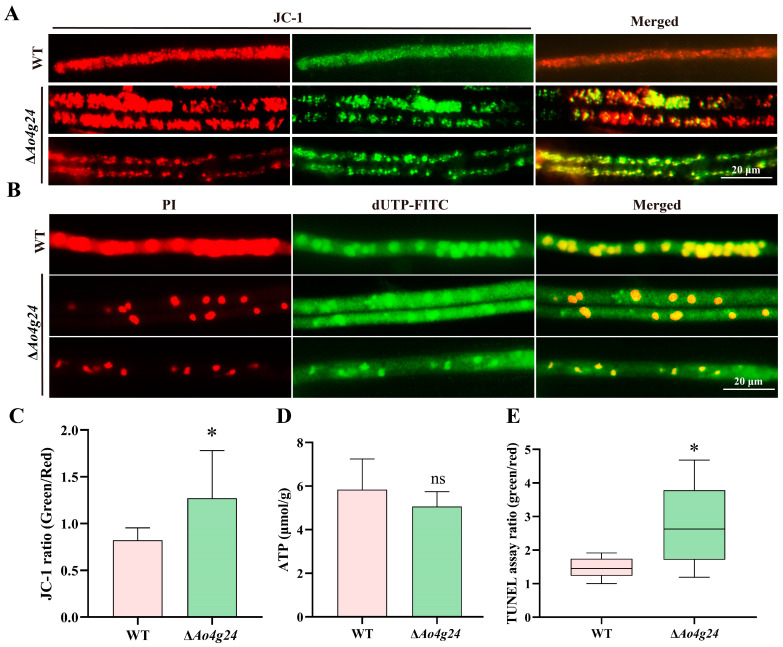
Roles of *Ao4g24* in MMP and apoptosis. (**A**) JC-1 staining of MMP in WT and Δ*Ao4g24* mutants. (**B**) TUNEL test in WT and Δ*Ao4g24* mutants. Green fluorescence indicates damaged DNA, whereas normal cell nuclei were labeled with red fluorescence. (**C**) Statistical analysis of MMP levels. The green to red FI value indicates the level of MMP reduction. (**D**) Comparison of ATP content. (**E**) Apoptosis analysis. The green to red FI value indicates the degree of cell apoptosis. A significant difference is denoted by an asterisk (* *p* < 0.05), ns indicates there is no significant difference between WT and Δ*Ao4g24* mutants.

In addition, we further evaluated mitochondrial activity in the Δ*Ao4g24* mutant strains by staining with JC-1 dye. The hyphae of the Δ*Ao4g24* mutant strains showed an increased green FI compared to the WT strain (Figure 6A). The green to red FI was obviously higher in the Δ*Ao4g24* mutant strains (Figure 6C). This was an indicator of the reduced MMP. Furthermore, the deletion of the *Ao4g24* gene led to a slight reduction in ATP content compared to the WT strain (Figure 6D). Additionally, the mutation of *Ao4g24* exhibited an obviously increased green FI relative to the WT strain in the dUTP-FITC staining. Statistical analysis of the green/red (dUTP-FITC to PI staining) FI showed a remarkably increased level of apoptosis in the hyphae of the Δ*Ao4g24* mutants compared to the WT strain (Figure 6E). These results indicate that *Ao4g24* affects the apoptosis of mycelia and may regulate mitochondrial activity.

### 3.7. Ao4g24 Regulates ROS Production and Stress Response

The ROS level of the WT and Δ*Ao4g24* mutants was detected through DHE and NBT dye staining. The hyphae of the Δ*Ao4g24* mutant strains had a higher ROS accumulation compared to the WT strain in the DHE staining (Figure S3A). Moreover, the FI levels of DHE in the WT and Δ*Ao4g24* mutant strains were 14.5 and 20, respectively (Figure S3C). Consistent with this, the mycelia of the Δ*Ao4g24* mutant strains exhibited a more pronounced staining with the NBT dye compared to those of the WT strain (Figure S3B), suggesting an increased ROS level in the Δ*Ao4g24* mutant strains. To determine the sensitivity of the *Ao4g24* response to external stimuli, we treated the WT and Δ*Ao4g24* mutant strains with different concentrations of oxidants (H_2_O_2_ and menadione) (Figure S4A) and osmotic pressure reagents (NaCl and sorbitol) and different temperatures (34–42 °C) (Figure S5A). The results showed that the ability of the Δ*Ao4g24* mutants to tolerate oxidative stress reagents was increased, as the RGI values were obviously lower than those of the WT strain (Figure S4B). However, the Δ*Ao4g24* mutants showed increased RGI values under 0.2 M NaCl, 0.5 M sorbitol, and 28 °C conditions (Figure S5B). In addition, *Aogld* and *Aoglr* (two genes related to oxidative stress) were significantly downregulated in the Δ*Ao4g24* mutants (Figure S4C), while the genes associated with osmotic stress (*Aohog1*, *Aoshol*, *Aosln1*, *Aossk1*, and *Aomsn2*) were remarkably downregulated in the Δ*Ao4g24* mutants. These results revealed the involvement of *Ao4g24* in ROS accumulation and stress response.

### 3.8. Deletion of Ao4g24 Impairs Secondary Metabolism

**Figure 7:**
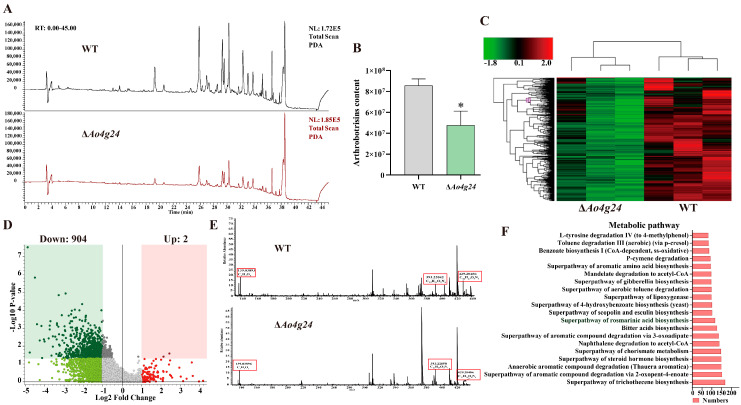
Comparison of metabolites in WT and ∆*Ao4g24* mutant strains. (**A**) Analysis of HPLC-MS. (**B**) Comparison of arthrobotrisins content. (**C**) Heatmap analysis. (**D**) Volcano plot analysis of differential metabolites. (**E**) Mass spectrogram of arthrobotrisins. (**F**) Top 20 metabolic pathways related to differentially expressed compounds. A significant difference between ∆*Ao4g24* mutants and WT strain is denoted by an asterisk (* *p* < 0.05).

High-performance liquid chromatography–mass spectrometry (HPLC-MS) analysis showed that the metabolic abundance of Δ*Ao4g24* mutant strains was not significantly different from that of the WT strain during a retention time (RT) of 16–40 min (Figure 7A). However, the specific metabolite arthrobotrisins of *A. oligospora* were detected during the retention time at 35.01 min, and the concentration of arthrobotrisins in the Δ*Ao4g24* mutant strains was remarkably reduced, as determined via a calculation of the peak area of the arthrobotrisins (Figure 7B). Afterward, the differential metabolites were further analyzed using clustering. The clustered heatmap exhibited a notable difference between the WT and Δ*Ao4g24* mutant strains (Figure 7C). The volcano plot analysis revealed that there were 907 downregulated and 2 upregulated compounds in the Δ*Ao4g24* mutant strains (Figure 7D). In addition, differential metabolic pathways were analyzed in the Δ*Ao4g24* mutant strains. The top 20 pathways associated with differentially expressed compounds are listed in Table S3. The highly enriched metabolic pathways were trichothecene biosynthesis, aromatic compound degradation, acetyl-CoA degradation, and amino acid biosynthesis (Figure 7F).

## 4. Discussion

Previous studies have identified that multiple genes are involved in trap formation in *A. oligospora*. However, some uncharacterized genes are also important for hyphal differentiation and the development of the trap structure. Here, we found that an unidentified gene, *Ao4g24*, is upregulated at trap formation (24 h); thus, it is assumed that *Ao4g24* may have an important impact on trap formation. Bioinformatic and functional analyses showed that Ao4g24 is specific to NT fungi but has a similar amino sequence to Snf5 of the SWI/SNF chromatin remodeler. Additionally, we revealed that *Ao4g24* contributes to sporulation, trap formation, and pathogenicity in *A. oligospora*. The SWI/SNF chromatin complex utilizes the energy derived from ATP hydrolysis to promote chromatin access, thereby regulating multiple cellular processes, and it plays essential roles in governing gene expression, hyphal development, and various stress responses [33]. Snf5 serves as the core component of the SWI/SNF complex. It is highly conserved, mostly found in the nucleus, and can be relocated to the cytoplasm in response to hypoxic circumstances [34]. The functions of the Snf5 homologous proteins have been identified in many fungal species, and Snf5 plays essential roles in regulating hyphal growth, conidiation, virulence, stress response, and metabolic balance [13,18,19]. Consistent with this, our study revealed that *Ao4g24* is involved in conidiation, trap formation, stress response, and secondary metabolism in *A. oligospora*, suggesting the conserved function among filamentous fungi. *Ao4g24* is dispensable for mycelial growth in *A. oligospora*, whereas *snf5* is important for hyphal development in other filamentous fungi, indicating the functional difference between *Ao4g24* and *snf5*.

The asexual conidiation is a prevalent method of reproduction in filamentous fungi and has a crucial impact on determining the survival and pathogenicity of fungal pathogens [35]. *A. oligospora* can generate conidia for asexual reproduction, and the spore is the most important component of the biocontrol agent [2]. Studies have shown that the knockout of *Mosnf5* results in a severe defective conidia morphology and decreases the conidiation in *M. oryzae* [13]. In concordance with this, the deletion of *Ao4g24* led to a dramatic decrease in conidia yield. Correspondingly, the transcription levels of several genes related to sporulation (including *AofluG*, *AovelB*, *AoabaA*, *AobrlA*, *AowetA*, *AoveA*, *AoflbC*, and *AonsdD*) were significantly downregulated in the Δ*Ao4g24* mutants. The central regulatory components, consisting of *abaA*, *brlA*, and *wetA*, have been reported to be indispensable for sporulation, conidial maturation, asexual development, and virulence in several model fungi, including *A. oligospora* [35], *A. nidulans* [36], and *B. bassiana* [37]. *fluG* is a significant inducer of the initiation of sporulation in *A. nidulans* [38], whereas the velvet complex *velB* connects the light-responding signal and regulation of sexual development [39]. In addition, *Casnf5* is a crucial determinant for mycelial growth; the mutant of *snf5* leads to the abnormal scenario of mycelia invasion in *C. albicans* [18], and *snf5* is also important for hyphal development in *Cryptococcal neoformans* [40]. However, the absence of *Ao4g24* does not affect the hyphal development. These results suggest that *Ao4g24* plays a different role in hyphal growth and conidiation.

The trap formation is a vital symbol of the NT fungi lifestyle transition from saprophytic to predacious, and multiple processes are involved in the process of trap formation, including cell cycle, signal transduction, and energy metabolism [4]. Additionally, the extracellular protease secreted by *A. oligospora* can be a virulence factor to infect and dissolve the nematodes [41,42]. The disruption of *Ao4g24* led to a dramatic reduction in the number of traps at 12, 24, 36, and 48 h, and the nematode predation ability was significantly reduced in the Δ*Ao4g24* mutant strains. However, the extracellular proteolytic activity of Δ*Ao4g24* mutants showed a slight increase relative to the WT strain, suggesting the increased ability of Δ*Ao4g24* mutants to digest the nematodes. Furthermore, previous studies have reported that several volatile compounds produced by NT fungi were closely related to the regulation of trap formation, such as 2-methyl-1-butanol (MB), methyl 3-methyl-2-butenoate (MMB), 6-methyl-salicylic acid (6-MSA), and arthrobotrisins [43,44,45]. In our metabolome analysis, the deletion of *Ao4g24* led to a significant reduction in many of the metabolic compounds shown in the volcano analysis, such as the content of arthrobotrisins, followed by a marked decrease in trap formation. In addition, a large number of aromatic degradation and acetyl-coA degradation compounds were significantly enriched in our metabolic pathway. Aromatic compounds are decomposed into several core intermediates via multiple metabolic pathways and function as an energy source in the TCA cycle [46]. Acetyl-CoA is a pivotal metabolic intermediate that controls multiple critical cellular processes, including energy metabolism, histones, and chromatin modification [47,48]. The trap formation requires a continuous energy supply [49]. In this study, the dramatic reduction in trap formation in the Δ*Ao4g24* mutants may be due to the deficiency in energy metabolism. From the combination of these results, it can be seen that *Ao4g24* plays a critical role in trap formation, pathogenicity, and the biosynthesis of secondary metabolites in *A. oligospora*.

The demand for energy and its maintenance is particularly important for trap formation and responding to various biotic and abiotic stimuli [49]. Mitochondria, which are multifunctional organelles, are mainly responsible for ATP production and supply within eukaryotic cells. Mitochondria play a crucial role in the regulation of multicellular processes, including autophagy, apoptosis, metabolic homeostasis, signal transduction, and ROS removal [50,51]. Mitochondria are the main site of ATP production, through mitochondrial oxidative phosphorylation [52]. In our results, the mutation of *Ao4g24* led to an enlarged volume and increased number of mitochondria, and the significantly decreased MMP indicated impaired mitochondrial function, which resulted in a slight reduction in ATP levels. Additionally, mitochondria have a central role in apoptotic cell death, and the MMP decline is a hallmark of the early cell apoptosis [51]. In the Δ*Ao4g24* mutants, the apoptosis was further evaluated with FITC-dUTP staining, and the degree of apoptosis was significantly increased. Taken together, these results suggest an essential role of *Ao4g24* in the regulation of mitochondrial function and cell apoptosis.

In addition to playing a role in ATP generation and apoptosis, autophagy, and ROS generation are regulated by mitochondrial respiration [53,54]. ROS have been considered as byproducts of cellular metabolism and could function as signal molecules that participate in multiple cellular pathways [55,56]. Damaged mitochondria are major producers of ROS, including the superoxide (O_2_^−^) anion, hydrogen peroxide (H_2_O_2_), the reactive hydroxyl radical (OH^-^), and autophagy, which are key players in the removal of damaged organelles and ROS accumulation [56]. Furthermore, ROS also have a signaling role in the regulation of autophagy. Highly elevated ROS generation leads to the oxidation of the mitochondrial inner membrane, resulting in impaired mitochondrial function and increased autophagy of damaged organelles [57]. In our findings, determined using DHE and NBT staining, the deletion of *Ao4g24* significantly increased the ROS level of the hypha, and the autophagosome was obviously increased in the Δ*Ao4g24* mutants. In agreement with this result, the expressions of several genes related to autophagy (*atg1*, *atg8*, *atg9*, and *atg13*) were significantly upregulated. *atg8* has been identified as playing a role in the regulation of key autophagy processes and as essential for cellular growth [58], sporulation [59], and appressorium formation [60]. In addition, several studies provide evidence that ROS production regulates autophagy to some extent [57,61]. These results showed the involvement of *Ao4g24* in the regulation of ROS production and autophagy.

Oxidative stress is characterized as an imbalance between ROS generation and the antioxidant defense system [62]. Stress responses are essential for NT fungi to sense and adapt to environmental changes. In our findings, we found that the Δ*Ao4g24* mutants were sensitive to low concentrations (5 mM) of oxidants (H_2_O_2_) and two different concentrations of osmotic stress reagents (0.1, 0.2 M NaCl and 0.25, 0.5 M sorbitol). However, the inactivation of *Ao4g24* resulted in a reduced RGI value in high concentrations of oxidants. Consistent with this, there are several antioxidant compounds that are enriched in the metabolic pathway, such as rosmarinic acid and aromatic compounds. Previous studies have reported that rosmarinic acid could serve as a powerful antioxidant by activating multiple pathways [63], and aromatic compounds have been used as antioxidants in medicine and food [64]. In addition to being sensitive to chemical stress reagents, the Δ*Ao4g24* mutants revealed an increased sensitivity to 38 °C conditions, but the tolerance to 42 °C was significantly increased. These results show that *Ao4g24* plays essential roles in response to stress stimuli. Importantly, heat stress is also thought to be an environmental factor that stimulates ROS production. The activation of ROS may be related to heat stress [65]. Furthermore, H_2_O_2_, one of the byproducts of O_2_ reduction, has been proven to induce the transcriptional activation of heat shock genes [66]. These results indicate that *Ao4g24* is crucial for stress response.

Here, we explored the role of an unknown gene, *Ao4g24*, in the NT fungus *A. oligospora*, including hyphal growth, conidiation, trap formation, stress response, and secondary metabolism. Our results showed that *Ao4g24* is critical for ROS generation induced by oxidative stress and heat shock and that it performs essential roles in energy metabolism, including autophagy, mitochondria, and apoptosis. On the one hand, the knockout of *Ao4g24* resulted in enlarged mitochondrial morphology and impaired mitochondrial function, and it increased autophagy, apoptosis, and ROS level; on the other hand, the loss of *Ao4g24* deteriorated the conidia yield, trap formation, and secondary metabolism. Therefore, our study highlights the importance of *Ao4g24* in sporulation, trap formation, and pathogenicity, and provides a reference for the exploration of the roles of unknown genes in NT fungi.

## Data Availability

All data generated or analyzed during this study are included in the published paper and associated Supplemental Files.

## Supplementary Materials

The following supporting information can be downloaded at: https://www.mdpi.com/article/10.3390/microorganisms12081532/s1, Figure S1: Differentially expressed genes associated with chromatin organization at trap formation (0 h, 24 h) in transcriptome analysis of WT strain; Figure S2: Multiple sequence alignment of Ao4g24 from different fungi; Figure S3: Analysis of ROS accumulation; Figure S4: Comparison of stress response to oxidative reagents; Figure S5: Comparison of stress response to osmotic stress reagents and different temperature; Table S1: Primers used for gene knockout and verification in this study; Table S2: Primers used for RT-qPCR analysis in WT and ∆*Ao4g24* mutants; Table S3: The top 20 pathways associated with differentially expressed compounds.
