# Supplementary material for: Functional Characterization of Ao4g24: An Uncharacterized Gene Involved in Conidiation, Trap Formation, Stress Response, and Secondary Metabolism in Arthrobotrys oligospora
Source: Microorganisms. 2024 Jul 26;12(8):1532. doi: 10.3390/microorganisms12081532 (PMC11356499; doi:10.3390/microorganisms12081532)
Supplement: Supplementary file 1 [file microorganisms-12-01532-s001.zip › microorganisms-3138051-supplementary.pdf]

Supporting Information

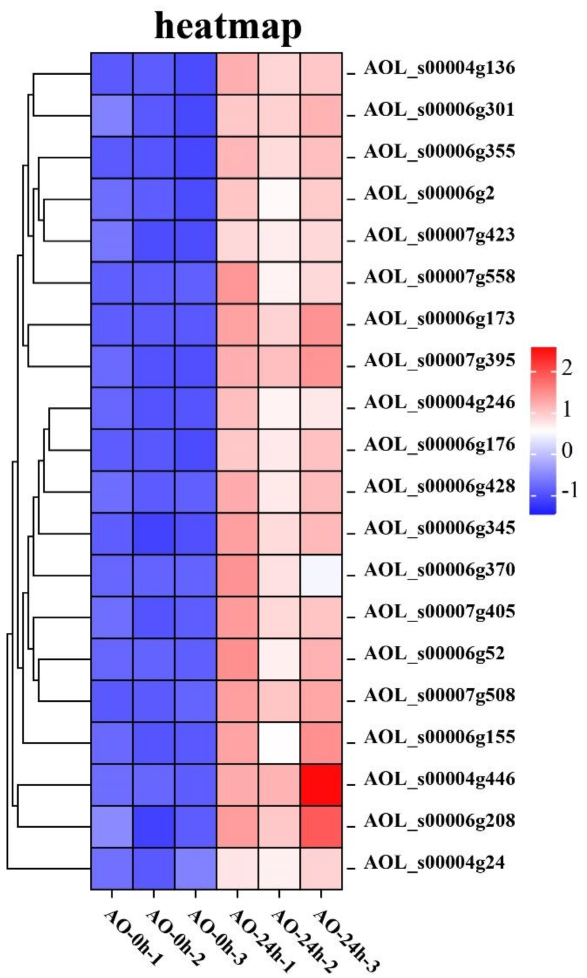

Figure S1. Differentially expressed genes associated with chromatin organization at trap formation (0 h, 24 h) in transcriptome analysis of WT strain. Ao4g24 is significantly downregulated at 0 h and upregulated at 24 h.

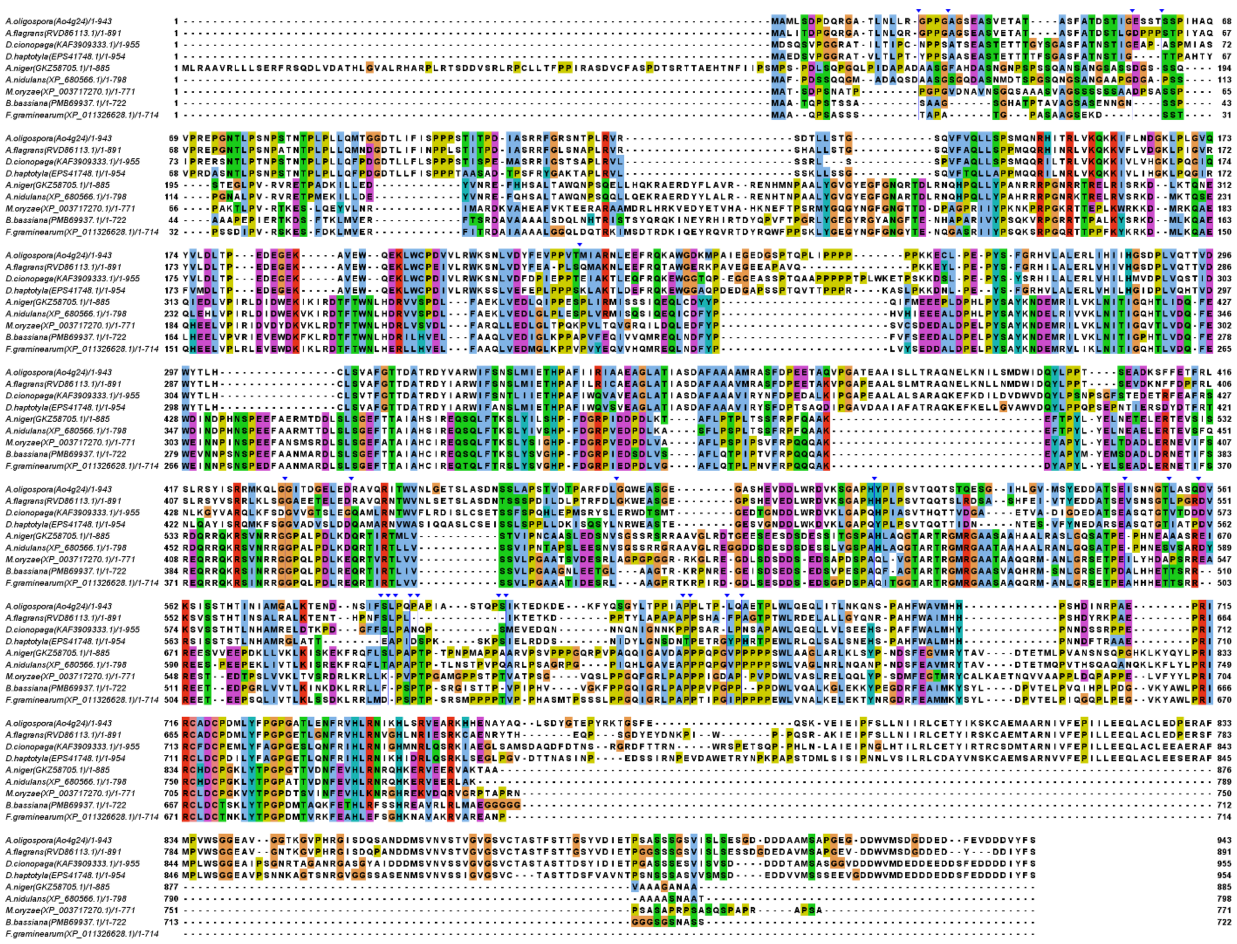

Figure S2. Multiple sequence alignment of Ao4g24 from different fungi. The amino acid sequences of Ao4g24 from different fungi are aligned by Jalview software.

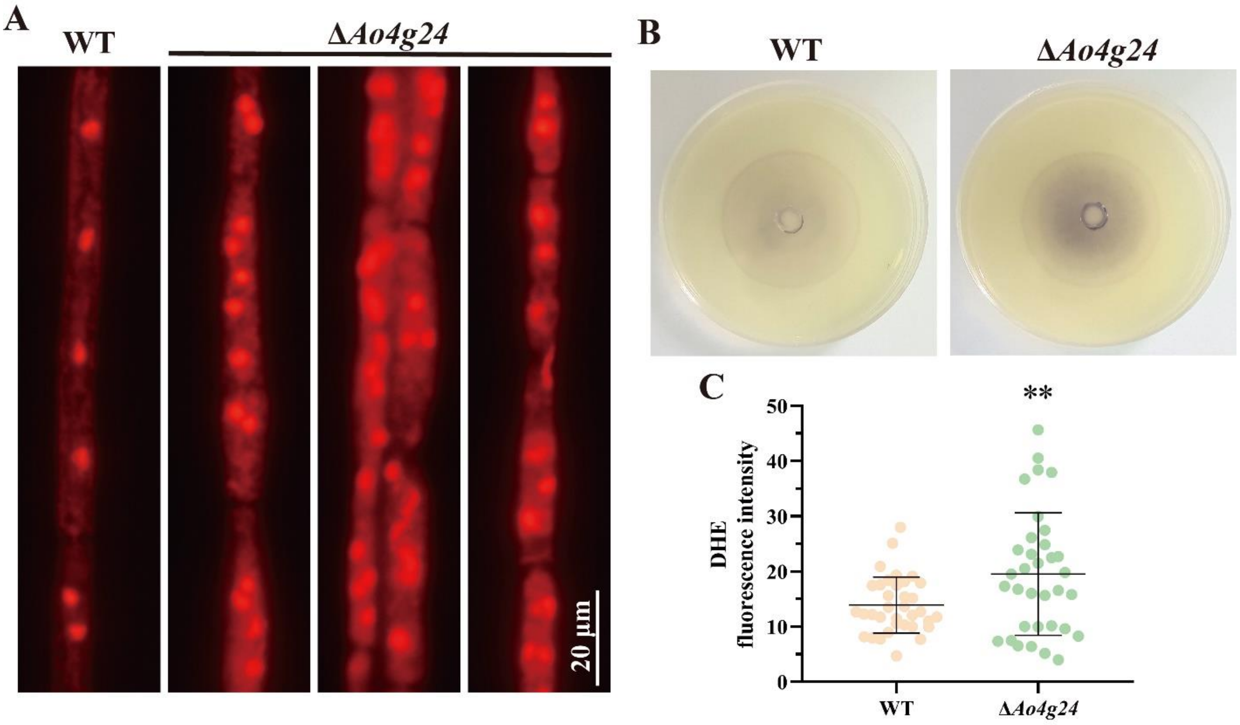

Figure S3. Analysis of ROS accumulation. **(A)** Observation of superoxide using DHE staining. **(B)** Analysis of ROS level via NBT staining. **(C)** Statistical analysis of DHE FI values. Significant difference between WT and Δ*Ao4g24* mutants is denoted by asterisk (*$p < 0.05$).

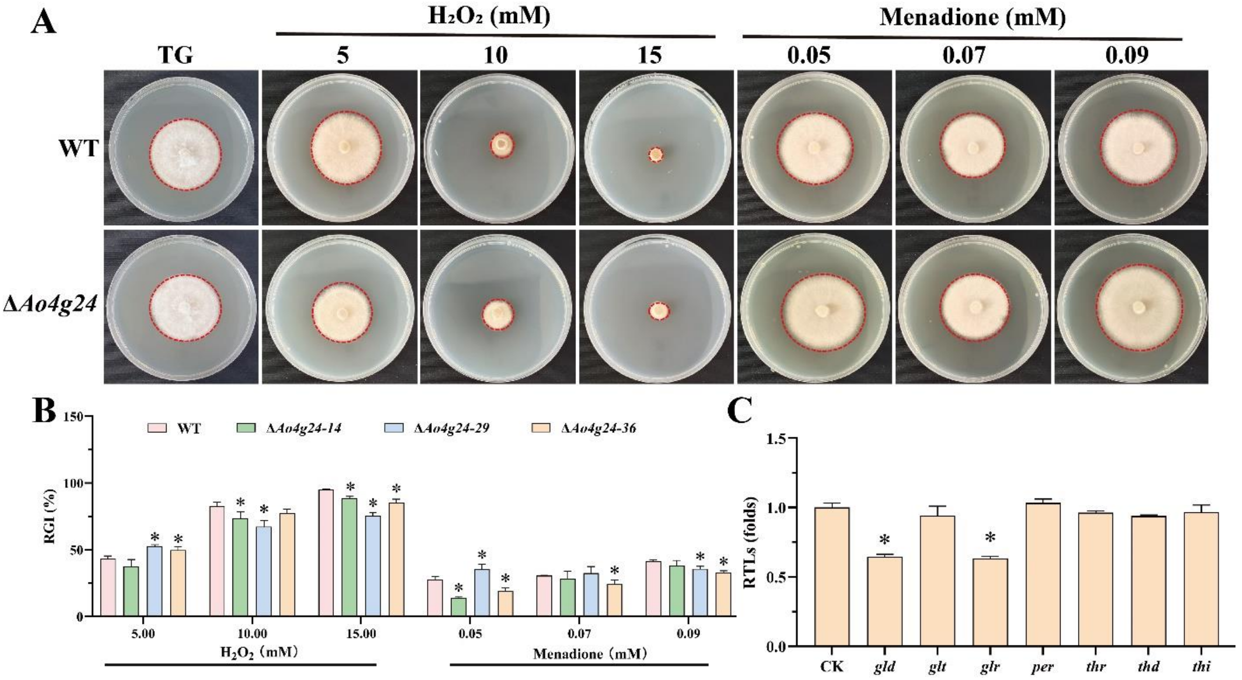

Figure S4. Comparison of stress response to oxidative reagents. **(A)** Colony morphology of WT and Δ*Ao4g24* mutant strains on TG medium supplemented with three different concentrations of oxidants (H_2O_2 and menadione). **(B)** Relative growth inhibition (RGI) values of WT and Δ*Ao4g24* mutant strains grown on corresponding medium. **(C)** Relative transcription levels (RTLs) of genes related to oxidative stress between the WT and Δ*Ao4g24* mutant strains. Significant difference between WT and Δ*Ao4g24* mutants is denoted by asterisk (*$p < 0.05$).

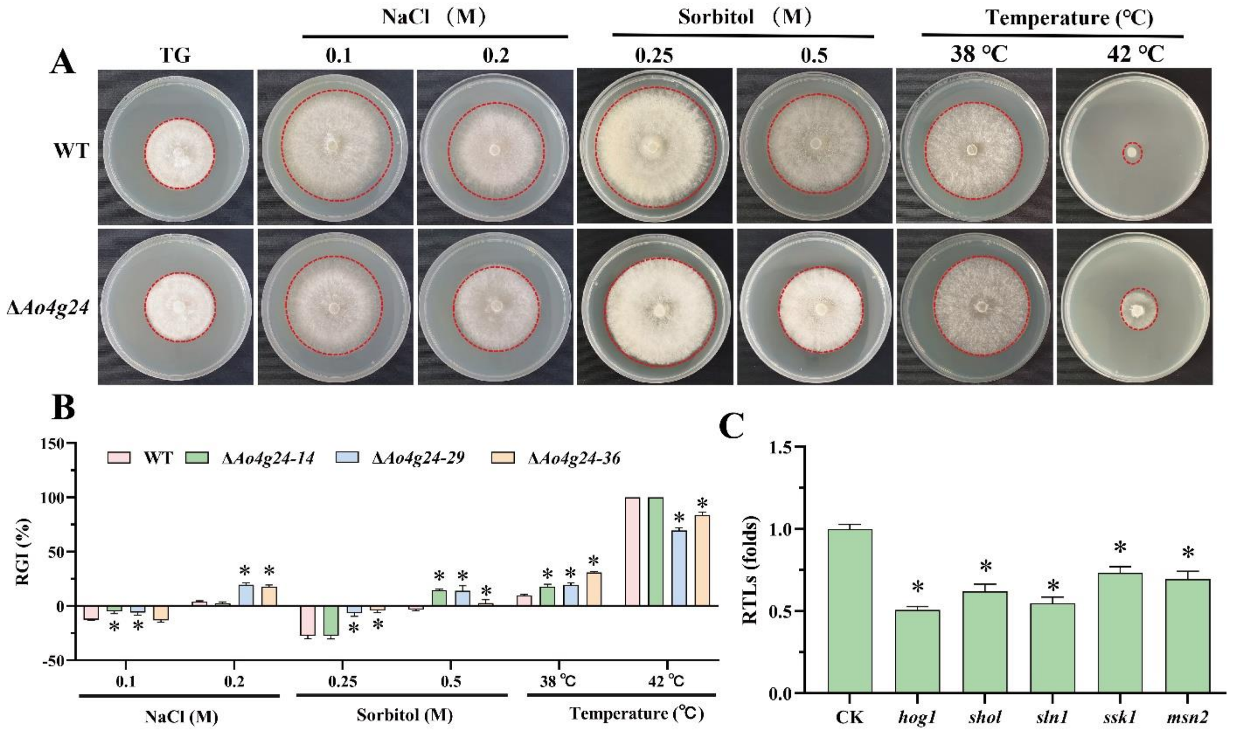

Figure S5. Comparison of stress response to osmotic stress reagents and different temperatures. **(A)** Colony morphology of WT and Δ*Ao4g24* mutant strains grown on TG medium supplemented with different concentrations of osmotic stress reagents (NaCl and sorbitol) and different temperature conditions. **(B)** RGI of WT and Δ*Ao4g24* mutant strains grown on corresponding meida. **(C)** RTLs of genes related to osmotic stress between the WT and Δ*Ao4g24* mutant strains. Significant difference between WT and Δ*Ao4g24* mutants is denoted by asterisk (*p < 0.05).

Table S1. Primers used for gene knockout and verification in this study.

	Sequence (5'-3')	Purpose
Ao4g24-5F	GTAACGCCAGGGTTTTCCCAGTCACGACG TCTGCGGACGGGACTTTA	Amplify the 5′ fragment
Ao4g24-5R	ATCCACTTAACGTTACTGAAATCTCCAACTTGAACGGAGGCGGATGA	
Ao4g24-3F	CTCCTTCAATATCATCTTCTGTCTCCGACGCCATATTTCACATAACTCC	Amplify the 3′ fragment
Ao4g24-3R	GCGGATAACAATTTCACACAGGAAACAGC TCACTGCTTGTCCACGAC	
hph-F	GTCGGAGACAGAAGATGATATTGAAGGAGC	Amplify the *hph* cassette
hph-R	GTTGGAGATTTCAGTAACGTTAAGTGGAT	
YZ-5F	GTGCCATAGCATCAAAGT	Transformants verification via PCR
YZ-3R	ACGACCGAGATGAACTAA	
RT-5F	CGACTATTTTGAGGTCCCACCA	Transformants verification via RT-qPCR
RT-5R	TGGTGGAGGAGGAGGGATTAAT	

Table S2. Primers used for RT-qPCR analysis in WT and Δ*Ao4g24* mutants.

Sporulation-related genes	Sequence (5F)	Sequence (3R)
AOL_s00043g361 (*fluG*)	GATTCCAGTCCCGTGAATTC	GCTAAGGAGAGGATGGGCAT
AOL_s00054g811 (*velB*)	ATTCCGCAACTTCTCCCTCA	GGCATGTTTGGATTCTGGGG
AOL_s00080g63 (*abaA*)	AACTTTATGCGCCTTGTCGT	TTGGCTAGGTGGTCTGTACG
AOL_s00097g514 (*brlA*)	TTGAGGCCTCGATCCGTAGA	AGGTAGATGGCGCTGTTACG
AOL_s00173g221 (*wetA*)	TTACATGCCACCCCAAGTCC	CAATTGCAACTGCGTCCACA
AOL_s00169g18 (*veA*)	AAGCTACACCCAATCAACGC	TTGCGATGCTGACGATCTTG
AOL_s00007g157 (*flbC*)	CTCTCCGGCAAAGACAATC	GTCGACTGAGGATAGTAGCT
AOL_s00075g211 (*nsdD*)	ATTACGGCCGCCTAGTAGTC	CTCGTTTGGACCTGGTTGTG
AOL_s00006g570 (*hyp1*)	GCGGATCCAACATGAAGCTT	GGTTGACAACTGGGATGCTG
**Autophagy-related genes**	**Sequence (5F)**	**Sequence (3R)**
AOL_s00076g234 (*atg1*)	GCCATTAGATTTGCCACCAG	CAGTTCCGTTCGTCACTCCC
AOL_s00007g534 (*atg8*)	AGCGTATCAAGCTGTCTCCC	CTCGTAGCCGAAGGTGTTTT
AOL_s00054g371 (*atg9*)	TTCTTCGGAGGACAGAGCAT	CTAGGTTTGCGTGAAAGTCG
AOL_s00215g74 (*atg13*)	AGAGGTGGAGGGTTGATTTA	GGAGTTTCCATGATGGCAGT
AOL_s00043g575 (*atg17*)	GAGATTCAGAACTCGTGGTT	AAGAAGTGAGGGTGTATTGC
**Genes related to oxidative stress**	**Sequence (5F)**	**Sequence (3R)**
AOL_s00043g396 (*gld*)	TTTGGATGTGGTGCTGTTGG	CTGTCGCCTCAATGATCGTG
AOL_s00054g242 (*glt*)	GGCCCCAGACTTCTCCCAGA	ACCGTTCCCCTTAAACTCGT
AOL_s00054g13 (*glr*)	CGAAAAGTCTGAATCGGGTGA	GCTCCACTTTGCCACATACATC
AOL_s00215g326 (*per*)	CACCATCCGCTCTGTCTTCA	GACGGCATCCTCGGTCTTGA
AOL_s00054g257 (*thr*)	TTTCGGTCAAGAGCAAAAGTG	GCTATCATTCCATTCCCCATT
AOL_s00078g209 (*thd*)	TGTCATCGGTGGTGGAGATT	ACAAGAGAGGTAGCGGGTTC
AOL_s00076g213 (*thi*)	TTGATTTCCACGCAACCTGG	CCGAACCTGATGAAGAAGCG
**Genes related to osmotic stress**	**Sequence (5F)**	**Sequence (3R)**
AOL_s00109g23 (*hog1*)	GAGGTCGATATCTGGAGTGCAG	TTTGGGTAACGACTGGACGAAT
AOL_s00078g396 (*shol*)	ACCGCGCAAAGGCTATTTATTC	GCAATACCTGTTTCGCCAGTTT
AOL_s00078g537 (*sln1*)	AGGGATCTCGTTACCCAGGTTA	AGTCGCCTCTCAAAATCCCATT
AOL_s00112g14 (*ssk1*)	GGAAGGGAAGGTGGCTCAAATA	GGGCGGTGATATCCTGATCATT
AOL_s00076g216 (*msn2*)	ACCCCAGTTGAGATTAAGCCAG	TGGGAGGAAAGACTCGACATTG
AOL_s00076g640 (*β-tublin*)	CCACCTTCGTCGGTAACTC	TCGTCCATACCCTCACCAG

Table S3. The top 20 pathways associated with differentially expressed compounds.

Metabolic Pathway	Compounds in Pathway
Superpathway of trichothecene biosynthesis	168
Superpathway of aromatic compound degradation via 2-oxopent-4-enoate	152
Anaerobic aromatic compound degradation (Thauera aromatica)	149
Superpathway of steroid hormone biosynthesis	149
Superpathway of chorismate metabolism	147
Naphthalene degradation to acetyl-CoA	139
Superpathway of aromatic compound degradation via 3-oxoadipate	135
Bitter acids biosynthesis	127
Superpathway of rosmarinic acid biosynthesis	117
Superpathway of scopolin and esculin biosynthesis	102

Superpathway of 4-hydroxybenzoate biosynthesis (yeast)	101
Superpathway of lipoxygenase	99
Superpathway of aerobic toluene degradation	98
Superpathway of gibberellin biosynthesis	97
Mandelate degradation to acetyl-CoA	97
Superpathway of aromatic amino acid biosynthesis	95
P-cymene degradation	93
Benzoate biosynthesis I (CoA-dependent, ss-oxidative)	86
Toluene degradation III (aerobic) (via p-cresol)	84
L-tyrosine degradation IV (to 4-methylphenol)	82
